# Impaired fin regeneration and angiogenesis in aged zebrafish and turquoise killifish

**DOI:** 10.1242/bio.059622

**Published:** 2023-04-07

**Authors:** Johanna Örling, Katri Kosonen, Jenna Villman, Martin Reichard, Ilkka Paatero

**Affiliations:** ^1^ Turku Bioscience Centre, University of Turku and Åbo Akademi University, FI-25020 Turku, Finland; ^2^ Institute of Vertebrate Biology, Czech Academy of Sciences, 60365 Brno, Czech Republic; ^3^ Department of Ecology and Vertebrate Zoology, University of Łódź, 90136 Łódź, Poland

**Keywords:** Zebrafish, Danio rerio, Turquoise killifish, Nothobranchius furzeri, Aging, Wound healing, Angiogenesis, VEGF, Regeneration, Fin

## Abstract

Impaired wound healing is associated with aging and has significant effects on human health on an individual level, but also on the whole health-care sector. Deficient angiogenesis appears to be involved in the process, but the underlying biology is still poorly understood. This is at least partially being explained by complexity and costs in using mammalian aging models. To understand aging-related vascular biology of impaired wound healing, we used zebrafish and turquoise killifish fin regeneration models. The regeneration of caudal fin after resection was significantly reduced in old individuals in both species. Age-related changes in angiogenesis, vascular density and expression levels of angiogenesis biomarker VEGF-A were observed. Furthermore, the anti-angiogenic drug vascular endothelial growth factor receptor blocking inhibitor SU5416 reduced regeneration, indicating a key role for angiogenesis in the regeneration of aging caudal fin despite aging-related changes in vasculature. Taken together, our data indicate that these fish fin regeneration models are suitable for studying aging-related decline in wound healing and associated alterations in aging vasculature.

## Teaser

**Summary:** Zebrafish and turquoise killifish are complementary models for aging studies. Aging reduced angiogenesis in regenerating caudal fin and anti-angiogenic pharmacological treatment reduced regeneration of aged caudal fin.

## INTRODUCTION

Aging is a feature affecting most, if not all, contemporary human populations. Aging, however, causes many physiological and biological changes in the individual and is also considered as one of the primary risk factors for complicated wound healing (Sgonc and Gruber, 2013). Challenged wound healing or chronic wounds are sometimes called a silent epidemic and they create a significant burden to affected individuals and also to the health-care sector (Sen et al., 2009). In developed countries the treatment expenses due to chronic wounds cover up to 5.5% of the healthcare costs (Posnett and Franks, 2008; Phillips et al., 2016).

Wound healing consists of four overlapping phases called hemostasis, inflammation, proliferation and remodeling, and complications in any of these phases contribute to poor wound healing (Frykberg and Banks, 2015). One of the crucial processes during wound healing is angiogenesis. Angiogenesis is required for tissue remodeling, and it initiates rapidly after wounding (Honnegowda et al., 2015). Angiogenic factors, such as VEGF, promote the action of endothelial cells for instance by stimulating their migration and proliferation (Li and Eriksson, 2001; Hellberg et al., 2010). Hypoxia plays a significant role in wound healing (Sammarco et al., 2015) and hypoxia inducible factor 1 (HIF-1α) acts as a stimulus for the production of VEGF (Acker and Plate, 2003). As wound healing enters the final phases, high levels of growth factors start to decrease, hypoxia subsides and angiogenesis is suppressed (Kumar et al., 2009). During aging, impaired HIF-1α signaling indicates a diminished response to hypoxia and thus plays a role in the delays concerning angiogenesis (Rivard et al., 2000). In addition, increased secretion of proinflammatory mediators due to the dysfunction of macrophages impairs the production of VEGF in the elderly (Ashcroft et al., 2002). Interestingly, impaired VEGF signaling has been suggested to play a key role in the aging process (Grunewald et al., 2021).

Although the underlying etiologies behind chronic wounds vary, they share many similarities, such as senescent cells unable to proliferate, dysfunctional stem cells, high levels of proteases and reactive oxygen species, increased levels of proinflammatory cytokines, and lack of microvasculature (Frykberg and Banks, 2015). Lack of microvasculature may result from poor angiogenesis, and angiogenesis is compromised during aging although the underlying mechanisms are largely not understood (Hodges et al., 2018).

Similarly to angiogenesis (Hodges et al., 2018), the mechanisms of wound healing have been studied in great detail in young individuals, but the defects in biological processes resulting in poor wound healing in aged individuals are still unclear in many ways (Goswami et al., 2021). One reason for this may be the long lifespan of laboratory mammals, which complicates aging studies. On the other hand, short-lived invertebrates lacking blood vessels may have limitations in potential to translate the findings into mammalian and human systems. In recent years, laboratory fish have been used as models for wound healing and tissue regeneration. Most commonly used model fish, zebrafish (*Danio rerio*), has fairly long lifespan but another shorter-lived model species, turquoise killifish (*Nothobranchius furzeri*), is gaining ground as suitable vertebrate model for aging studies (Harel and Brunet, 2015).

Here, we have used zebrafish and turquoise killifish as complementary models to study aging-related decline in wound healing by using fin regeneration model. We have especially focused on the alterations of angiogenic signaling and vasculature associated with compromised fin regeneration in this study.

## RESULTS

### Declined regeneration of caudal fin in aged zebrafish

**Fig. 1.**
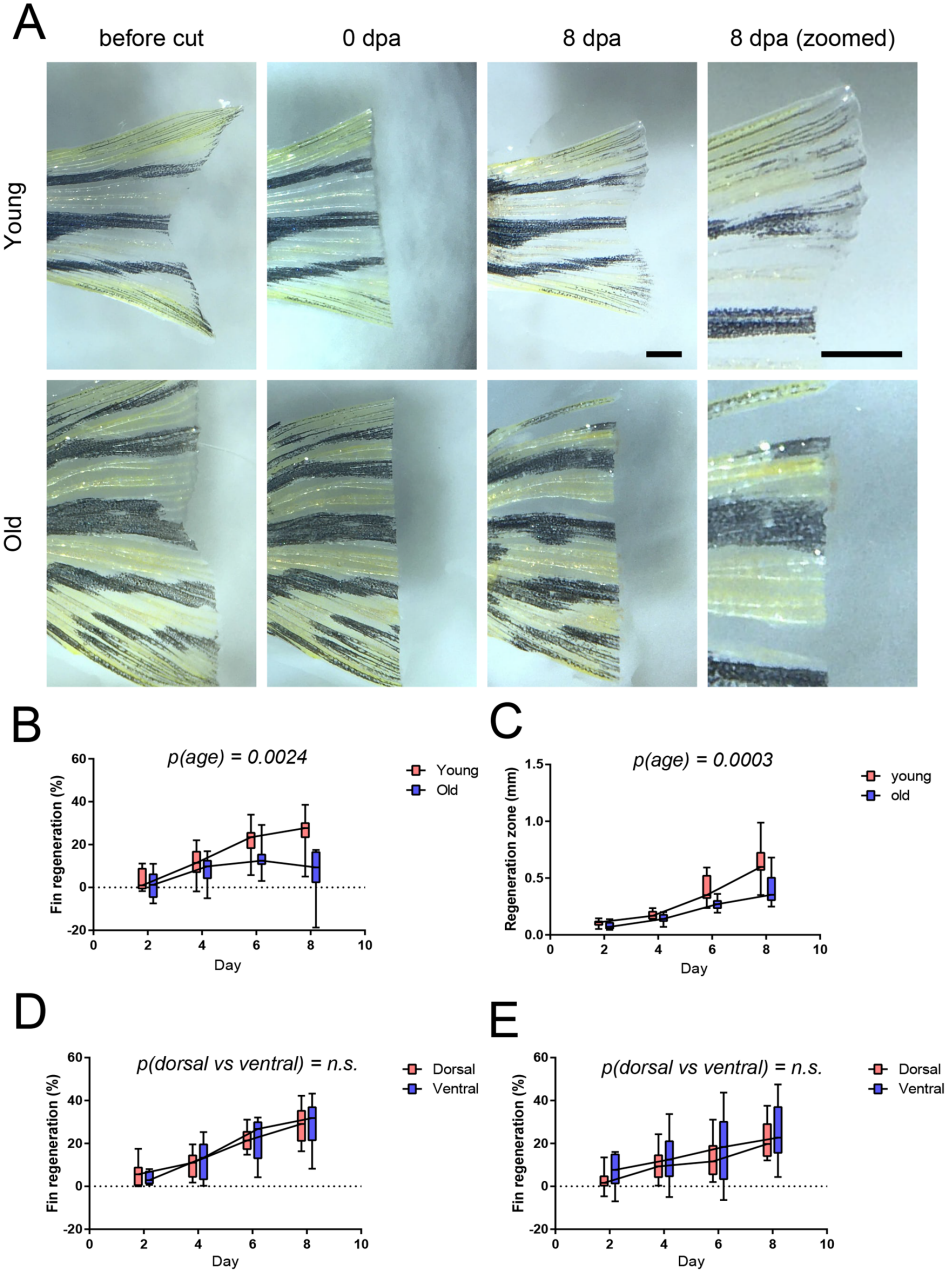
**Aging reduces the regeneration of zebrafish caudal fin.** Old (4 years) and young (4 months) male zebrafish were subjected to caudal fin regeneration assay. (A) Microscopy images of fins before, right after and 8 days after resection. Scale bar: 1 mm. (B) Quantification of the total regeneration of the caudal fin. Two-way ANOVA with age and time as factors was used for statistical analysis. All time points were used in the statistical analysis. (C) Quantitation of thickness of regenerating area. Two-way ANOVA with age and time as factors was used for statistical analysis. All time points were used in the statistical analysis. (D,E) Comparison of the regeneration in dorsal and ventral part of caudal fin. Data from young fish in panel D and in old fish in E. *n*=12 in both age groups. Two-way ANOVA, with age and anatomical location as factors, was used for statistical analysis.

To gain insights into age-associated decline in fin regeneration we raised zebrafish to very old age (40 months). Then, we carried out a zebrafish caudal fin regeneration assay using both old (40 months) and young (4 months) zebrafish (Fig. 1A). Interestingly, the old fish had clearly diminished capability to regenerate resected caudal fins. Both the total regeneration of caudal fin (Fig. 1B) and the thickness of hypopigmented regenerating area (Fig. 1C) were reduced in aged individuals. The regeneration of both dorsal and ventral part of the caudal fin were similarly reduced in aged individuals (Fig. 1D) indicating proper morphological control of regeneration.

### Declined regeneration of caudal fin in aged turquoise killifish

**Fig. 2.**
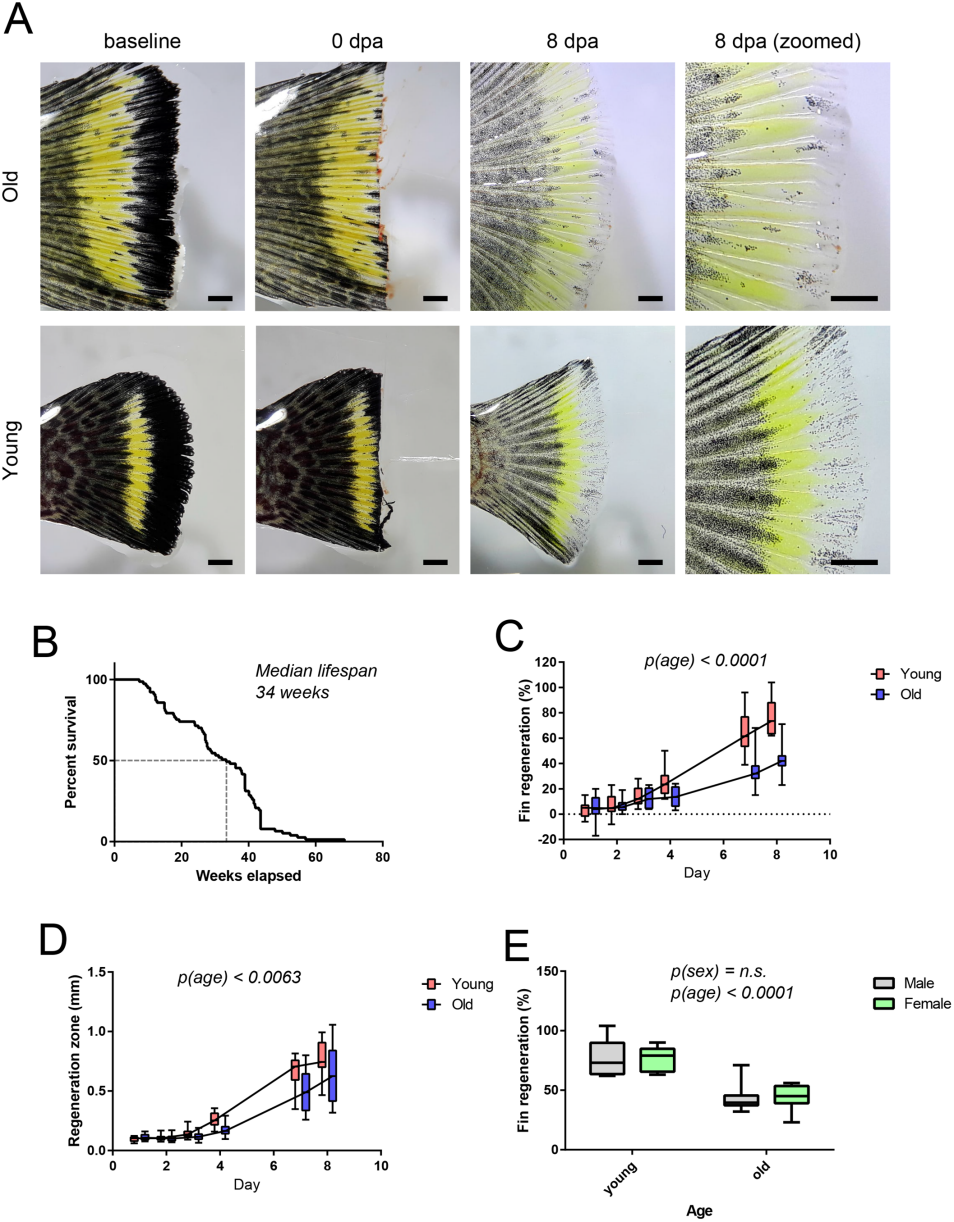
**Aging reduces the regeneration of turquoise killifish caudal fin.** Old and young turquoise killifish of both sexes were subjected to caudal fin regeneration assay. In total four groups were used (young male, old male, young female, old female). Young were 5 weeks of age and old 12 weeks when entering the experiment. (A) Microscopy images of turquoise killifish caudal fins before, immediately after and 8 days after resection. Scale bar: 1 mm. (B) Kaplan–Meier survival curve of turquoise killifish strain MSCZ222 housed in Turku, Finland. *n*=77. (C) Quantification of the total regeneration of the caudal fin. Two-way ANOVA with age and time as factors was used for statistical analysis, *n*=8 per group. All time points were used in the statistical analysis. (D) Thickness of regenerating zone. Two-way ANOVA with age and time as factors was used for statistical analysis. All timepoints were used in the statistical analysis. (E) Comparison of the total regeneration in male and female turquoise killifish caudal fin. In E, two-way ANOVA with sex and age as factors was used for statistical analysis. *n*=8 per group.

Zebrafish is a considerably long-lived small fish species and obtaining adequately old zebrafish is a slow process. To overcome these limitations, the short-lived turquoise killifish (*Nothobranchius furzeri*) has been proposed as a suitable fish model for aging studies (Harel and Brunet, 2015). Therefore, we established a turquoise killifish colony of strain MZCS-222 (Blažek et al., 2013) in fish facility in Turku, Finland, to carry out caudal fin regeneration experiments in turquoise killifish. The median lifespan of killifish is known to vary between facilities. In the Turku facility the MZCS-222 strain of turquoise killifish had a median lifespan of 34 weeks (Fig. 2B), which is considerably shorter than the approximately 160-week median lifespan of zebrafish (Gerhard et al., 2002). Caudal fin regeneration experiments were carried out in a similar manner as with zebrafish, with young killifish being 5 weeks and killifish 12 weeks of age (Fig. 2A). Indeed, both the total fin regeneration (Fig. 2C) and thickness of regeneration zone (Fig. 2D) of resected caudal fins of killifish was already reduced in 12-week-old animals compared to 5-week-old young adult fish. These observations were consistent with earlier observations made with a longer-lived killifish strain MZM-0703 (Wendler et al., 2015).

There were no differences between the sexes as both males and females displayed similar growth rates and similar decline of regeneration to old individuals (Fig. 2E). To our knowledge, this is the first observation addressing potential sex differences in fin regeneration in turquoise killifish. In zebrafish, the sex of the animals is not always documented during wound healing and fin regeneration experiments. However, some evidence implies that in general sex differences could be small or absent during regeneration of zebrafish caudal fin, but may also differ in other tissues such as pectoral fins (Nachtrab et al., 2011).

### The vascular density increases in aged individuals

**Fig. 3.**
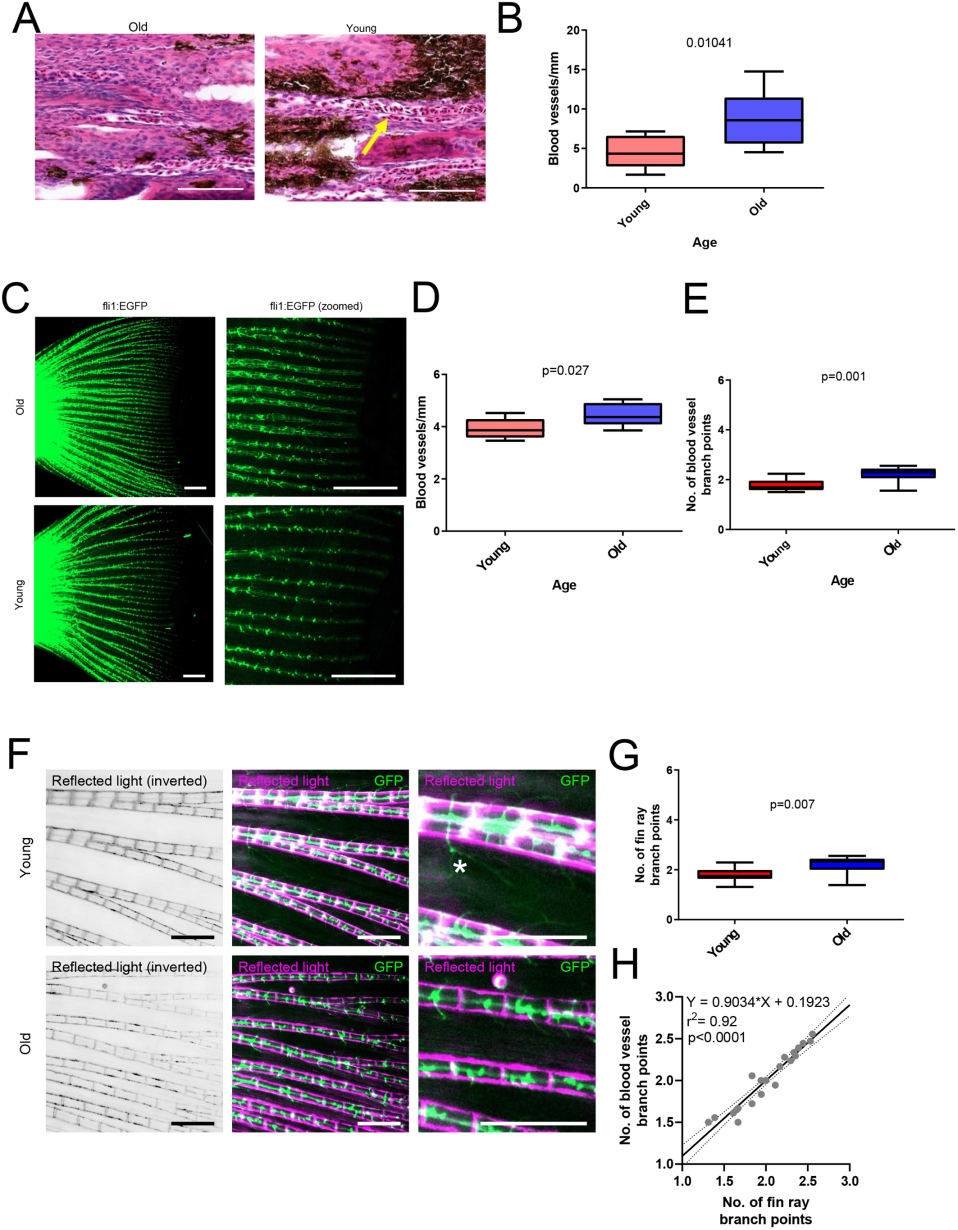
**Aging increases vascular density in caudal fins.** (A) Images of blood vessels in histological section of old and young turquoise killifish caudal fins stained with Hematoxylin and Eosin. An example of a blood vessel is indicated with a yellow arrow. Scale bar: 100 µm. (B) Quantification of blood vessel density in turquoise killifish sections. Statistical analysis with Mann–Whitney *U*-test, *n*=8 per group. (C) Fluorescence images of vasculature of transgenic zebrafish [*roy, mitfa, Tg(fli1:EGFP)*] caudal fins. Old fish were 36 months (*n*=8) and young fish 16 months (*n*=10). Scale bar: 1 mm. (D) Quantification of blood vessel density in transgenic zebrafish caudal fins. Statistical analysis with Mann–Whitney *U*-test. (E) Quantification of major blood vessel branch points in transgenic zebrafish caudal fins. (F) Fluorescence and reflected white light illumination images of transgenic zebrafish caudal fins. Scale bar: 0.5 mm. (G) Quantification of branching of fin rays. (H) Correlation analysis of fin ray and vascular branches. Statistical analysis using linear regression, both old and young samples combined, *n*=18.

To obtain deeper insights into the regeneration process, we analysed tissue sections of fins from old and young killifish. In histological analyses, we surprisingly observed increased blood vessel density in killifish fins during aging (Fig. 3A and B). The analysis of vasculature from these tissue sections was, however, quite challenging and we were able to detect only patent blood vessels occupied with red blood cells. To confirm observation of the age-related changes in vasculature and angiogenic potential, we analysed old and young transgenic zebrafish carrying EGFP in vascular endothelium [*roy, mitfa, Tg(fli1:EGFP)*] using fluorescence microscopy. This analysis indicated that the vascular density was increased also in zebrafish during aging (Fig. 3C and D). The number of branching points was increased in aged blood vessels (Fig. 3E), explaining the increased vascular density. In normal conditions, the caudal fin vasculature is largely organised along fin rays (Hlushchuk et al., 2016). Therefore, we analysed whether the increased vascular density was actually a result of increased branching of fin rays or from ectopic vascular branches. In the images of fin rays and vasculature (Fig. 3F), we observed similarly increased branching in fin rays (Fig. 3G), which was tightly correlated with increased vascular branching (Fig. 3H). This indicated that increased baseline vascular density is likely not a result of increased angiogenesis and formation of new ectopic vessels, but rather a secondary result of increased branching of fin rays.

### Angiogenic potential declines in aged fins

**Fig. 4.**
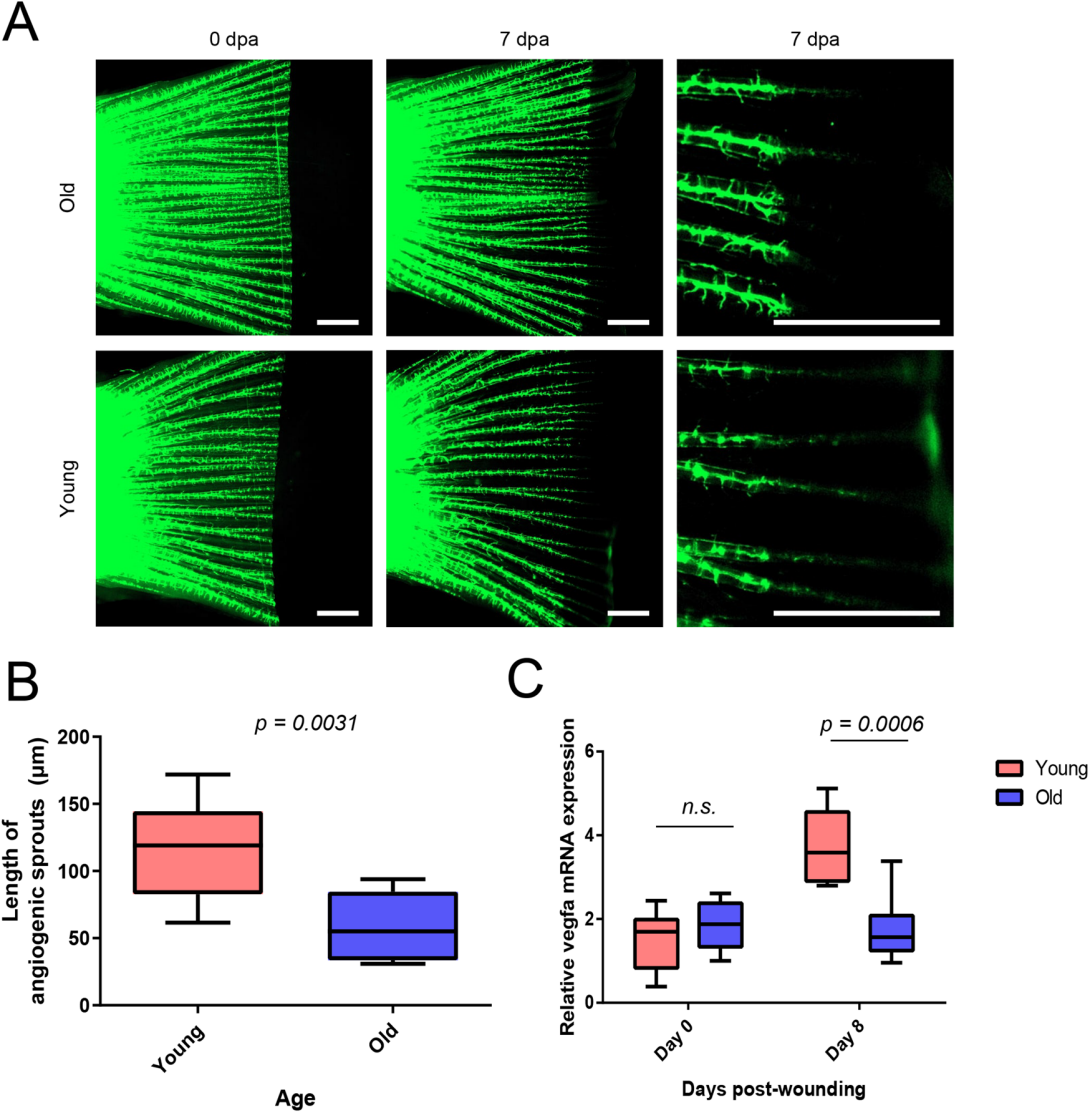
**Angiogenesis is reduced in aged regenerating caudal fins.** Zebrafish [genotype *roy, mitfa, Tg(fli1:EGFP)*] were subjected to caudal fin angiogenesis assay. Old fish were 36 months of age and young fish 16 months. (A) Fluorescence microscopy images of transgenic zebrafish caudal fins during fin regeneration experiment. Statistical analysis with Mann–Whitney *U*-test. (B) Quantification of angiogenesis in transgenic zebrafish caudal fins. Old fish (*n*=8), young fish (*n*=10). (C) qRT-PCR analysis of *vegfa* mRNA expression during fin regeneration assay in turquoise killifish. *n*=6 in each group. Statistical analysis was done with two-way ANOVA, age and time used as factors and multiple comparison testing with Sidak’s post-hoc test.

While closely examining the fluorescence microscopy images of young zebrafish (Fig. 3F), we identified some prominently long endothelial protrusions potentially indicative of active angiogenesis. Unfortunately, the obtained resolution of these images did not allow reliable quantitation of these smaller protrusions and only major vessels were reliably observed. To more directly analyse the capability for angiogenesis, we carried out zebrafish caudal fin angiogenesis assay (Hlushchuk et al., 2016) using transgenic zebrafish *roy, mitfa, Tg(fli1:EGFP)*. The distal part of the caudal fin was resected as before, and the regrowth of blood vessels was followed using fluorescence microscopy. Interestingly, the newly developed vascular sprouts during fin regeneration were shorter in aged fish (Fig. 4A and B). This indicated that the angiogenesis was impaired in aged zebrafish. Despite of higher density of blood vessels in aged fins, their angiogenic potential seemed to be diminished.

To further characterize changes in the vasculature, we analysed the expression of key angiogenic growth factor *vegfa* using qPCR. The expression of *vegfa* was induced in young regenerating fins, whereas this response was missing in old killifish (Fig. 4C).

These results indicated that alterations in vasculature and angiogenic *vegfa* signaling may underlie the aging-related decline in fin regeneration.

### Regeneration of aged caudal fin is inhibited by anti-angiogenic drug

**Fig. 5.**
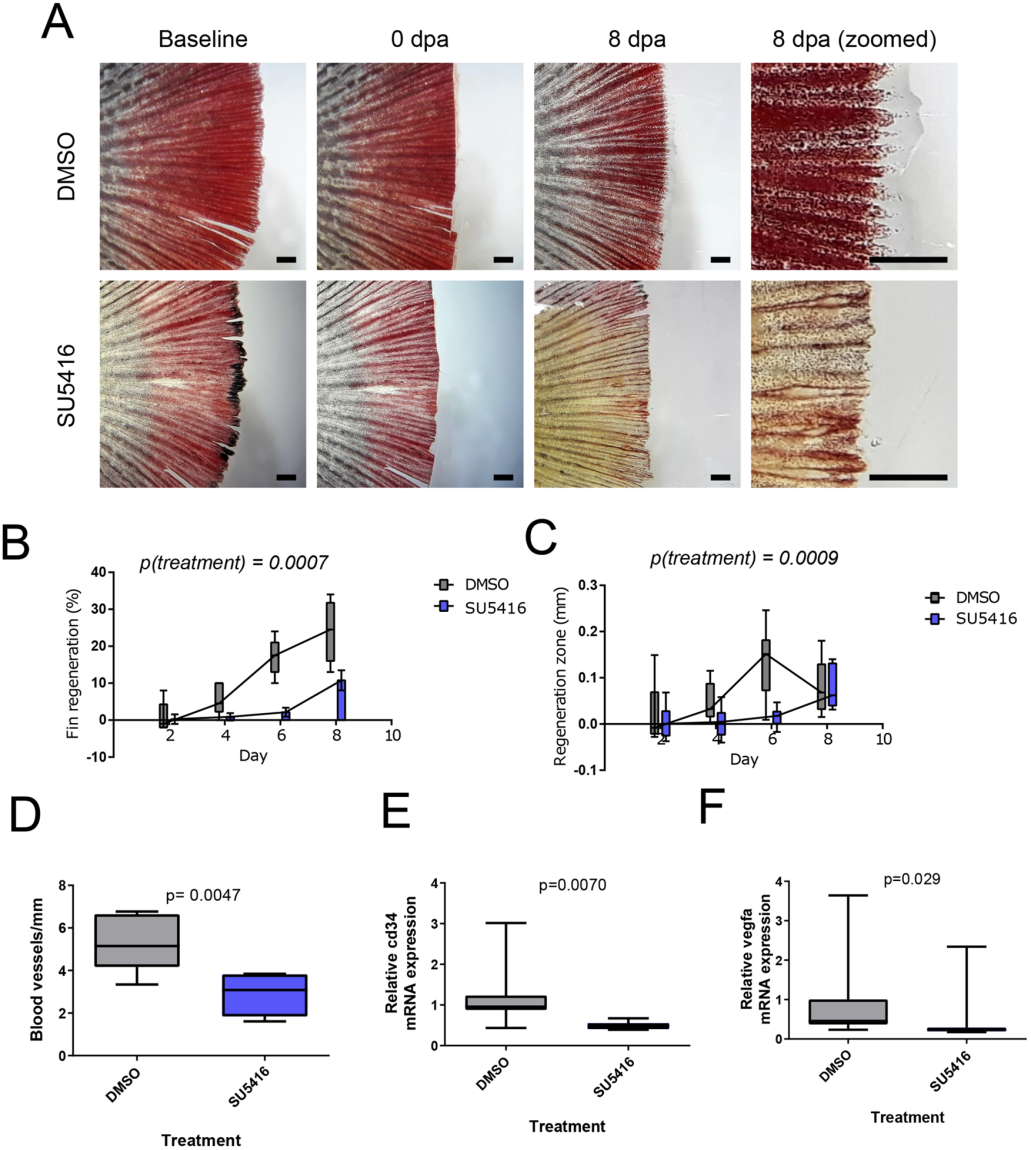
**Anti-angiogenic drug SU5416 reduces caudal fin regeneration in aged fish.** Caudal fin angiogenesis assay with old turquoise killifish (aged 32 weeks) and control or SU5416 treatment. (A) Microscopy images of caudal fins of turquoise killifish during caudal fin regeneration assay *n*=6 for DMSO and *n*=8 for SU5416. Scale bar: 1 mm. (B) Quantification of total regeneration of caudal fin. (C) Quantification of thickness of regenerating area. (D) Measurement of blood vessel density in histological sections. (E,F) qPCR measurements of mRNA expression of vascular biomarkers *cd34* (E) and *vegfa* (F). *n*=7 for both groups. Statistical analysis in C and D is two-way ANOVA (treatment and time as factors, all time points used in analysis) and in D, E and F Mann–Whitney *U*-test.

These data indicated that dysregulated angiogenesis may play a role in reduced regeneration of caudal fin in aged individuals. In young adult zebrafish, the fin regeneration is dependent on angiogenesis and pharmacological inhibition of VEGF signaling can reduce fin regeneration (Bayliss et al., 2006). To address whether VEGF signaling is still required in fin regeneration of aged fish, we treated the killifish with VEGF-receptor inhibitor SU5416 (Fong et al., 1999) after resection of the caudal fin. Indeed, this treatment reduced the growth of caudal fins in aged killifish (Fig. 5A,B,C). The observation of effectiveness of SU5416 to alter fin regeneration in aged killifish is in line with the earlier observations in young adult zebrafish (Bayliss et al., 2006). As expected, we observed also reduced number of perfused vessels in histological sections (Fig. 5D). To confirm this further, we analysed vascular biomarkers *vegfa* and *cd34* using qPCR. The reduced expression of vascular biomarkers *cd34* (Fig. 5E) and *vegfa* (Fig. 5F) indicated the effectiveness of anti-angiogenic SU5416 treatment in targeting vasculature.

### SU5416 alters vascular biomarkers, collagen deposition and cellular proliferation

**Fig. 6.**
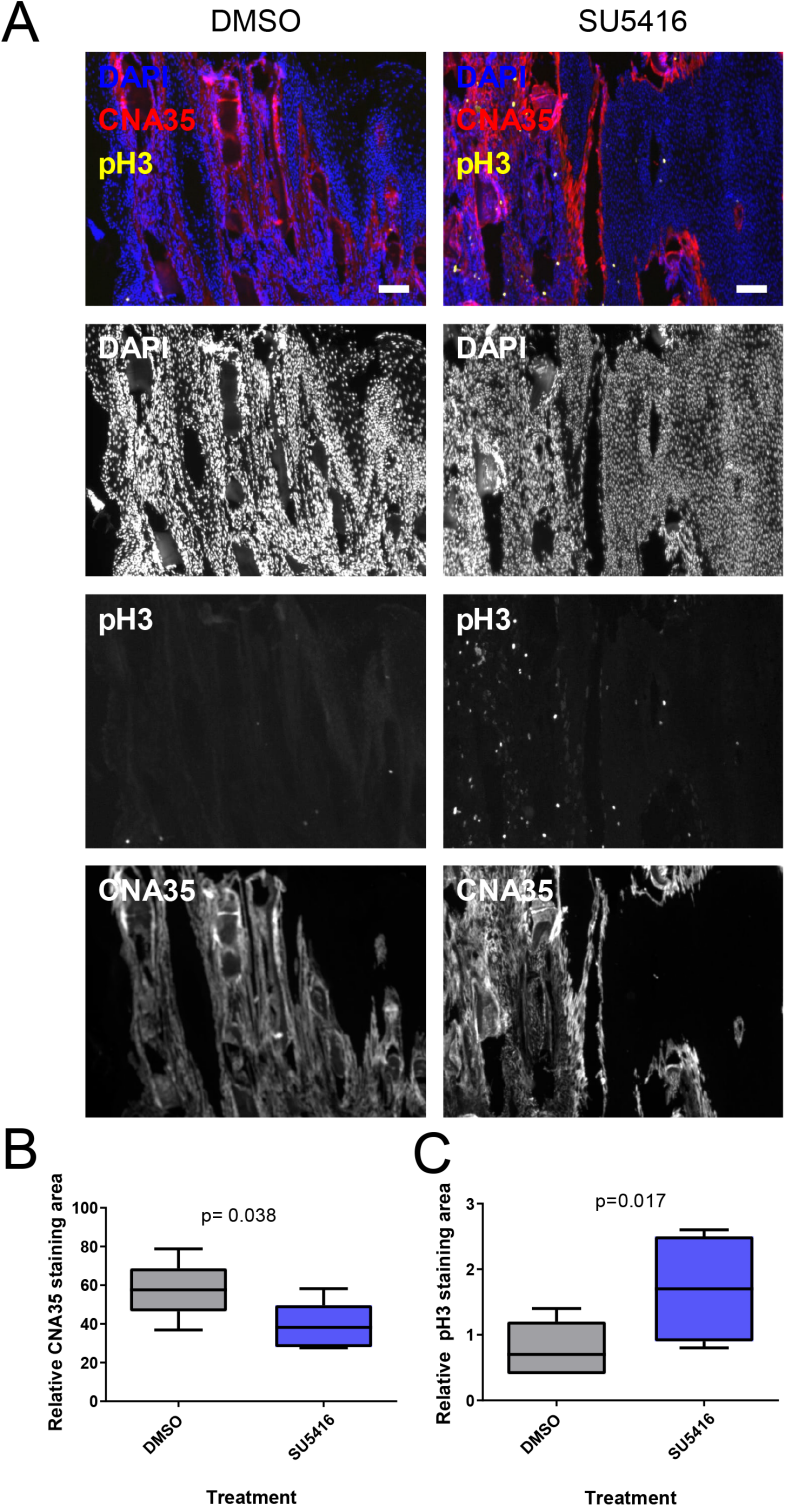
**Anti-angiogenic drug SU5416 alters cellular response during turquoise killifish caudal fin regeneration.** Samples were obtained from turquoise killifish (age 32 weeks) caudal fin regeneration assay and processed to histological sections. (A) Image of immunostaining for proliferation (pH3), collagen (CNA35) and nuclei (DAPI). Scale bar: 0.1 mm. (B) Quantification of collagen content stained by CNA-35 probe. (C) Quantification of cell proliferation marker phospho-histone 3 (pH3). *n*=7 in both groups.

To gain more in-depth analysis of tissue regeneration, we carried out immunostainings of regenerating fins of turquoise killifish (Fig. 6A). Interestingly, the amount of collagen was reduced (Fig. 6B) indicating defects in the regeneration of the caudal fin tissue structures. Abnormally high cell-proliferation rates were observed after SU5416 treatment (Fig. 6C), and these proliferating cells were localized throughout the fin tissue. This indicates that the effects of the inhibition of VEGF signaling extend beyond vascular signaling and angiogenesis in regenerating fins.

## DISCUSSION

In our experiments, we observed that aging results in declined fin regeneration in both zebrafish and turquoise killifish. This is in line with the earlier observations with fin regeneration model of turquoise killifish (Wendler et al., 2015). The previous results in zebrafish have been more controversial, and both decline and normal fin regeneration have been observed (Tsai et al., 2007; Itou et al., 2012). One explanation could be in the long lifespan of zebrafish. Zebrafish can live up to 5 years in laboratory, but 2-year-old fish are often considered middle-aged to old and begin to show signs of aging in aging studies (Kishi et al., 2009). Although aging is a gradual process and aging-related changes occur well before reaching maximal lifespan, the pace of aging varies across phenotypic traits (Hayward et al., 2015). Hence, using fish much closer to the actual maximal life span could provide clearer results. This, however, causes problems in carrying out the experiments due to long aging period before obtaining suitable populations.

Turquoise killifish is much more amenable to aging studies due to its shorter natural lifespan (Harel and Brunet, 2015). The fish strain MZCS-222 used in this study had median lifespan of 34 weeks when housed in Turku, Finland. It is also important to note that many factors in husbandry may affect lifespan as conditions in different laboratories may differ in numerous ways (Polačik et al., 2016). The same strain housed in Brno, Czech Republic, had a lifespan of 18-25 weeks. The factors underlying the variation involve temperature, diet, water quality parameters, frequency of mating and mycobacterial infection (Polačik et al., 2016; Reichard et al., 2022). Therefore, it is important to understand the aging dynamics also in the laboratory carrying out the experiments.

In our experiments, we focused on the earlier part of the fin regeneration since the onset and early stages have been found to be especially problematic in poorly healing wounds in human patients (Frykberg and Banks, 2015). We observed a delayed fin regeneration in our 8-day regeneration experiments. The full regeneration lasts approximately 28 days in young zebrafish (Hlushchuk et al., 2016) and turquoise killifish (Wendler et al., 2015), and in old fish the duration of full regeneration is likely to be longer. Therefore, we cannot tell whether the regenerating fins in the aging fish can complete the regeneration process or if there are any defective structures remaining due to incomplete regeneration. This is interesting topic to be followed in later studies.

The analysis of histological sections of killifish fins was technologically challenging and prone to human error, and therefore the key findings were confirmed by qPCR of vasculature and angiogenesis-related biomarkers and experiments using transgenic zebrafish. Development of endothelium-detecting antibodies working in killifish or novel transgenic turquoise killifish lines for vasculature research would significantly facilitate future studies on aging vasculature. During our experiments, we observed increased vascular density and controversially also a reduced angiogenic potential in aged fish. Increased vascular density was likely a secondary effect, as the vessels were tightly associated with fin rays that were more branched in aged fish. The reduced angiogenesis during fin regeneration was, however, functionally significant. The blockage of VEGF signaling had a significant effect on fin regeneration in aged turquoise killifish, indicating that despite reduced angiogenesis and VEGF expression, the VEGF signaling system remains functional and physiologically relevant in the caudal fins of aging fish. This observation is supported by earlier results of impaired VEGF signaling during the aging process in mammals (Grunewald et al., 2021). Our findings imply a central role for angiogenesis during various processes of fin regeneration extending beyond the growth of new blood vessels. Many of these effects are likely to arise secondarily due to the lack of functional vasculature to support new regenerating tissues with sufficient oxygen and nutrients.

Interestingly, poor angiogenesis has been implicated also with poor wound healing in humans (Cheng and Fu, 2018). This underlines the need to obtain deeper mechanistic understanding of the mechanisms of both angiogenesis and wound healing in aged tissues. The aging-related changes in angiogenesis are still inadequately characterized and there is a lack of suitable models to characterize them (Hodges et al., 2018). Our data indicate that the fin regeneration models of turquoise killifish and zebrafish may be suitable models to characterize the role of and mechanisms of angiogenesis in wound healing of aged animals. This can pave the way for future identification of suitable angiogenic agents with potential to enhance wound healing in aged individuals.

## MATERIALS AND METHODS

### Fish husbandry and ethics

The authorization for this project was obtained from the Animal Experimental Board of the Regional State Administrative Agency for Southern Finland (ESAVI/16458/2019 and ESAVI/2402/2021). The turquoise killifish in the facility were maintained according to protocol from Polačik et al. (2016) by the trained personnel and zebrafish according to standard procedures. Killifish were fed twice a day with frozen blood worms, and zebrafish were fed with Gemma micro dry food. Water parameters in turquoise killifish system were: temperature 26°C; salinity 230±30 µS and pH 7.3-7.6.

### Zebrafish caudal fin regeneration assay

Wild-type male zebrafish (AB line) aged 4 months (12 fish) and 4 years (12 fish) were used for studying the differences in fin regeneration between young and aged fish. At the beginning of the study (day 0), the fish were anesthetized with 200 mg/l tricaine methanosulfonate MS222 (Sigma-Aldrich) diluted in the system water. As the fish showed no physical reactions upon touching, the depth of the anesthesia was considered deep enough. The fish was placed on a petri dish lid with the help of tablespoon, and its caudal was spread carefully on top of the lid. After this, the caudal fin was photographed via Zeiss Stemi DV4 stereomicroscope using smart phone camera and an adapter. Finally, a small piece of distal caudal fin was resected with a scalpel, and the fin was photographed once more. Half of the resected piece of fin was stored at −80°C, and the other half was fixed in 10% neutral buffered formalin. After recovering from anesthesia, all the fish were housed individually in a 1 L tank, which contained 0.004% methylene blue in system water. The tanks were placed in a warm room (26°C) for the duration of the experiment. The system water containing Methylene Blue in the tanks was changed to fresh every other day and the fish were fed just before water change. During the study the fish were anesthetized at days 2, 4, 6 and 8, and their caudal fins were photographed under stereo microscope. At the end of the study (day 8), the regenerated part of distal caudal fin was again amputated under anaesthesia and finally stored at −80°C.

The thickness of the regeneration zone was measured from the resection plane to the distal end of the regenerating fin. The fin regeneration percentage was measured by dividing the thickness of the regeneration zone by the length from the resection plane to the distal end of intact caudal fin from images obtained prior to resection.

### Turquoise killifish caudal fin regeneration assay

Both genders of turquoise killifish (*Nothobranchius furzeri,* strain MZCS-222, Reichard lab, Institute of Vertebrate Biology, Czech Academy of Sciences, Brno, Czech Republic) was used. The experiment had eight fish in four groups: young male (age 5 weeks), young female (age 5 weeks), old male (age 12 weeks) and old female (age 12 weeks). First, the fish were anaesthetized with 400 mg/l of tricaine. The distal part of the caudal fin was resected under stereomicroscope (Zeiss Stemi DV4). The caudal fin was imaged before and after resection using a cell phone camera attached to stereomicroscope. The experiment was carried out as described above for zebrafish. The regrowth of fins was imaged on days 1, 2, 3, 4, 7 and 8. After the fish were fed with blood worms, the remaining food and debris was removed with a pipette and half of the water was replaced.

SU5416 (Santa Cruz Biotechnologies) or DMSO (Sigma-Aldrich) alone were dissolved in water. The fish (age 32 weeks) were bathed in drug solution, and 50% of the liquid was replenished daily 1 h after the feeding of the killifish. The fish were imaged at days 0, 2, 4, 6 and 8.

### Analysis of vascular density and branching in zebrafish caudal fin

Zebrafish [roy, mitfa, *Tg(fli1:EGFP)^y1^*] of younger adult population (age 16 months, 10 fish) and old adult population (age 36 months, 8 fish) were used in the experiment. The fins were manually fully spread along dorsoventral axis for imaging. For imaging, the zebrafish were anesthetized, transferred to a petri dish and imaged using Zeiss AxioZoom fluorescence stereomicroscope with total magnification of 16×. The microscope was equipped with 1.0x PlanApo Z objective, HXP 200C mercury lamp, Alexa 488, with the filter set at 38 HE, (Excitation: BP 470/40 nm, Emission: BP 525/50 nm) and Hamamatsu sCMOS Orca Flash4.0 LT+ camera. Reflected light images were captured by illuminating the samples with CL 9000 LED CAN ring light against black background and collecting the reflected light without a filter cube. The full range of image was used, and along anterior-posterior axis the measurement was carried out distally to last branch points close to distal edge of the caudal fin vasculature. In addition to vascular density, the number of branch points in each vessel were measured. Only major vessels were measured in the assay as capillaries were below our imaging resolution.

### Zebrafish caudal fin angiogenesis assay

The analysis of angiogenesis in zebrafish caudal fin was carried out largely similarly as fin regeneration assays. Zebrafish [*roy, mitfa, Tg(fli1:EGFP)^y1^*] of younger adult population (age 16 months, 10 fish) and old adult population (age 36 months, 8 fish) were used in the experiment. The fins were imaged at 3, 5 and 7 days post-amputation (dpa) and the experiment was ended at 7 dpa after imaging. For imaging, the zebrafish were anesthetized, transferred to a petri dish and imaged using Zeiss AxioZoom fluorescence stereomicroscope with total magnification of 16x. The microscope was equipped with 1.0x PlanApo Z objective, HXP 200C mercury lamp, Alexa 488, with the filter set at 38 HE, (Excitation: BP 470/40 nm, Emission: BP 525/50 nm) and Hamamatsu sCMOS Orca Flash4.0 LT+ camera. Reflected light images were captured by illuminating the samples with CL 9000 LED CAN ring light against black background and collecting the reflected light without a filter cube. Only major vessels were measured in the assay as capillaries were below our imaging resolution.

### RNA extraction and quantitative PCR

The RNA form resected fin pieces was extracted using NucleoSpin RNA Plus XS kit for RNA purification (Macherey-Nagel) according to the manufacturers protocol. The extracted RNA was stored at −80°C until subjected to reverse transcription using a High-Capacity cDNA Reverse Transcription Kit (Applied Biosystems). The obtained cDNA samples were stored at −20°C until analysed using qPCR.

Primer design for housekeeping gene *tbp* and selected angiogenesis-related genes *cd34* and *vegfa* was carried out using NCBI primer design tool (Ye et al., 2012). Primer sequences were: *tbp*(5′-AGCGTTTTGCTGCCGTCATA-3′, 5′-TTGACTGCTCCTCACTTTTGG-3′), *cd34*(5′-AGATGTGTGCTGCAAGGGCA-3′, 5′-CCTGTAACGTCATCTTCCACG-3′), and *vegfa*(5′-AAGCCGAGAAGATGAGAGCG-3′, 5′-TGTCAGACCAAGGGGAATGC-3′). Prior to PCR measurements, the cDNA samples were diluted to nuclease-free sterile water. Four replicate PCR reactions from each sample were prepared using PowerUp™ SYBR™ Green Master Mix (ThermoFisher Scientific), and sterile water was used as a negative control. All the reactions were performed on 96-well plates and analysed using QuantStudio 12kFlex instrument (Applied Biosystems). Raw data was further analysed with Relative Quantification application in ThermoFisher Cloud service (https://apps.thermofisher.com).

### Histology

The caudal fin pieces fixed in 10% neutral buffered formalin were dehydrated through an ascending series of alcohol, defatted and cleared in xylene and finally infiltrated and embedded to paraffin on a transverse plane. From the paraffin embedded samples, 4 µm sections were cut with the help of a microtome and the sections were collected to microscope slides. Before each staining, xylene was used for removing the paraffin from the sections, and the sections were also rehydrated in a descending series of alcohol. Samples were stained with Hematoxylin and Eosin according to standard protocol and mounted with Depex (Merck). Samples were imaged using Pannoramic P1000 slide scanner (3DHistech).

### Immunofluorescence stainings

The 4 µm sections cut form the paraffin embedded caudal fin pieces were double stained with anti-phospho-histone H3 (pH3, Cell Signaling Technologies, clone D2C8) and collagen-binding adhesion protein 35 (CNA35). To stain and detect the mitotic cells in the tissue, polyclonal rabbit phospho-histone 3 (pH3, ser10) antibody (Cell Signaling) together with Alexa Fluor 647 conjugated anti-rabbit secondary antibody (Invitrogen) were used. To stain collagen, CNA35 integrated to pET28a mCherry vector was used. pET28a-mCherry-CNA35 was a gift from Maarten Merkx (Addgene plasmid # 61607) (Aper et al., 2014). Molecular weight of the purified CNA35 was 60 kDa and the protein concentration was 7.65 mg/ml. The concentrations used in the staining were 1:200 for pH3, 1:1000 for secondary antibody and 1:100 for CNA35. For dilutions 10% FBS (Gibco) in PBS was used. Following deparaffinization and rehydration of the sections, antigen retrieval was carried out in Retriever 2100 (Aptum Bio) using Universal Buffer (10x, R Universal, Aptum Bio) diluted to the concentration of 1:10 with MilliQ water. Unspecific binding of the antibody in the sections was blocked by incubating the slides in 10% FBS for 1 h. Overnight incubation with primary antibody was done at +4°C and protected from light. Following this, the slides were washed with PBS and incubated with secondary antibody for 1 h protected from light at room temperature. DAPI diluted with PBS to the concentration of 0.002 mg/ml was used as a background stain. The slides were incubated with DAPI for 10 min in dark at room temperature. Finally, the slides were washed with PBS and mounted with water based Mowiol 4-88 (Calbiochem) mounting media, which also contained 2.5% DABCO (Sigma-Aldrich) as an antifading chemical.

Zeiss AxioZoom V16 fluorescence stereomicroscope with total magnification of 16x was used. The microscope was equipped with 1.0x PlanApo Z objective, HXP 200C mercury lamp, DAPI with the filter set at 49 (Excitation: G365 nm, Emission: BP 445/50 nm), Alexa 488 with the filter set at 38 HE, (Excitation: BP 470/40 nm, Emission: BP 525/50 nm), Alexa 568 with the filter set at 45 (Excitation: BP 560/40 nm, Emission: BP 630/75 nm), Alexa 647 - filter set 50 (Excitation: BP 640/30 nm, Emission: 690/50 nm) and Hamamatsu sCMOS Orca Flash4.0 LT+camera.

### Image processing

The image analysis and processing were carried out using free image analysis platforms FIJI (Schindelin et al., 2012) and QuPath (Bankhead et al., 2017). QuPath was used for calculating blood vessels in the tissue sections. Vascular density was analysed along the central part of the section along dorsoventral axis, and the correct anterior–posterior location was determined by observed fin ray structures. Immunofluorescence analyses were carried out in FIJI, and collagen area and mitotic cell area were calculated and measured using automatic thresholding. Also, the lengths of the regenerated caudal fins both in zebrafish and turquoise killifish studies were calculated in FIJI. The calculations were done both from the dorsal and ventral side of the fin. Some images were uploaded and handled on OMERO server (Allan et al., 2012). Publication images were produced using OMERO.figure and Inkscape. Images were linearly adjusted for brightness and contrast.

### Statistical analyses

Statistical analyses were carried out using GraphPad Prism (versions 6 and 9). Time-series were analysed with two-way ANOVA by using time as a factor and all time points in the analyses. Two-sample comparisons were done using non-parametric Mann–Whitney *U-*test. Correlation between fin ray and blood vessel branch points was analysed using linear regression. Prior to the experimentation, the appropriate sample size for caudal fin regeneration assay was obtained from power calculations using an online calculator (htp://powerandsamplesize.com/) using 2-groups, beta (power) 70% or 80% and alfa 5%. Estimated effect size in calculations was 50% and standard deviation 40% resulting in sample sizes of 8 (70% power) to 11 (80% power). Samples were neither formally randomized nor blinded during experimentation. Data were plotted as box-plots indicating median and 1st and 3rd quartiles, with whiskers extending from minimum to maximum.
